# A Review of Speech Perception of Mandarin-Speaking Children With Cochlear Implantation

**DOI:** 10.3389/fnins.2021.773694

**Published:** 2021-12-14

**Authors:** Qi Gao, Lena L. N. Wong, Fei Chen

**Affiliations:** ^1^Department of Electrical and Electronic Engineering, Southern University of Science and Technology, Shenzhen, China; ^2^Faculty of Education, The University of Hong Kong, Pokfulam, Hong Kong SAR, China

**Keywords:** cochlear implant, Mandarin, children, speech perception, outcome measures

## Abstract

**Objective:** This paper reviewed the literature on the development of and factors affecting speech perception of Mandarin-speaking children with cochlear implantation (CI). We also summarized speech outcome measures in standard Mandarin for evaluating auditory and speech perception of children with CI.

**Method:** A comprehensive search of Google Scholar and PubMed was conducted from March to June 2021. Search terms used were speech perception/lexical tone recognition/auditory perception AND cochlear implant AND Mandarin/Chinese.

**Conclusion:** Unilateral CI recipients demonstrated continuous improvements in auditory and speech perception for several years post-activation. Younger age at implantation and longer duration of CI use contribute to better speech perception. Having undergone a hearing aid trial before implantation and having caregivers whose educational level is higher may lead to better performance. While the findings that support the use of CI to improve speech perception continue to grow, much research is needed to validate the use of unilateral and bilateral implantation. Evidence to date, however, revealed bimodal benefits over CI-only conditions in lexical tone recognition and sentence perception in noise. Due to scarcity of research, conclusions on the benefits of bilateral CIs compared to unilateral CI or bimodal CI use cannot be drawn. Therefore, future research on bimodal and bilateral CIs is needed to guide evidence-based clinical practice.

## Introduction

In Western societies, the advantages of bilateral cochlear implantation (CI) over unilateral CI for speech perception in quiet and in noise, preverbal communication development and sound localization in the pediatric population have been well demonstrated (Sparreboom et al., 2010). The effects of adding a contralateral hearing aid (HA) among children implanted in the other ear (i.e., bimodal stimulation) have been demonstrated through extensive comparative studies as well (e.g., Beijen et al., 2008). However, unilateral CI is still the norm in mainland China, with the other two modes of amplification gaining popularity in the past decade only. With emerging research on this topic and the gradual reduction in the age of implantation, it is necessary to synthesize new evidence regarding speech perception of Mandarin-speaking children with unilateral CI, bimodal stimulation and bilateral CIs in order to guide clinical application and identify knowledge gaps. This review attempts to cover areas not addressed in the review by Chen and Wong (2017).

The first multi-channel CI operation was conducted in mainland China in 1995 (Liang and Mason, 2013). Since then, CI has become a well-accepted intervention for patients with severe-to-profound hearing loss (HL), funded by local and the central government, due to its cost-effectiveness compared to no intervention or HA (Qiu et al., 2017). Han and Wang (2013) reported over 30,000 persons in mainland China have received CIs, and among them 85% were children. In several provinces, unilateral CI for pediatric population is included in the basic medical insurance scheme (Li J. N. et al., 2017). Despite the fact that CI penetration in the pediatric population is less than 5% (Liang and Mason, 2013), the rate of implantation is expected to grow with the number of qualified specialists and hearing service providers (Li J. N. et al., 2017).

Unlike English, Mandarin is a tonal language with four lexical tones that carry lexical meaning at the monosyllabic level. Lexical tone recognition plays an important role in Mandarin sentence perception (Fu et al., 1998). Superior sentence recognition was noted in normal-hearing (NH) individuals listening to vocoded speech and pediatric CI users when sentences were presented with natural tone contours compared to flattened or randomized tones in quiet, and greater benefit was observed in noise, suggesting the importance of lexical tone contour (Chen F. et al., 2014; Huang et al., 2020). In addition, CI users needed a greater fundamental frequency (F0) range to detect lexical tones at a comparable level as NH listeners (He et al., 2016). Mandarin vowels also convey more intelligibility information than consonants in sentence perception in a ratio of 3:1 compared to 2:1 in English (Chen F. et al., 2013). Furthermore, Mandarin listeners relied more heavily on temporal fine structure when recognizing sentences in competing speech compared with English native listeners who rely more on temporal envelope (Wang et al., 2014). As CIs provide limited access to temporal fine structure and pitch information because of the coarse frequency resolution, it is reasonable to speculate that some findings regarding speech perception among English-speaking CI users may not apply directly to the Mandarin-speaking CI population. Thus, there is a need to synthesize evidence from studies that targeted this population.

Prior to the review, standard Mandarin speech outcome measures are summarized, highlighting their use and limitations. We then reviewed the current evidence related to speech perception with CI and factors influencing speech perception among pediatric users who speak Mandarin as their first language. Evidence on unilateral, bimodal, and bilateral CI use will be presented in separate sections.

## Method

Between March and June 2021, Google Scholar and PubMed were searched for relevant studies. The search terms were speech perception/lexical tone recognition/auditory perception AND cochlear implant AND Mandarin/Chinese. Due to the advancement of CI algorithms in the past two decades, we limited the search on publication year from 2000 onward. We focused on speech perception of participants with congenital HL, who spoke Mandarin as their first language and received CI. Only studies that were conducted in mainland China and published in English were included.

**FIGURE 1:**
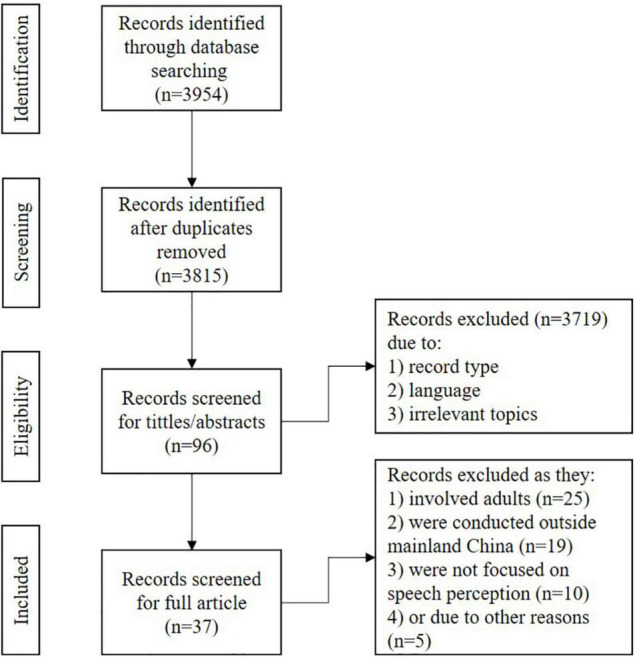
A flowchart of searching and screening.

The search generated a total of 3954 records relevant to the topic. After removing duplicates, 3815 records remained. After screening the titles and/or abstracts, 3719 records were discarded because they were not published in peer-reviewed journals, written in English and/or involved irrelevant content. Among the 96 articles that were retrieved for full-text screening, 58 articles were further excluded because results from children and adults were not reported separately (*n* = 25), the studies were conducted outside of mainland China (*n* = 19), the studies did not focus on speech perception (*n* = 10), findings from non-CI participants were not reported separately (*n* = 4), and only an abstract was available (*n* = 1). Finally, 37 articles remained for review. A flowchart of the screening process can be found in Figure 1. Among the 37 articles, 30 studies targeted Mandarin-speaking children with unilateral CI, and 5 studies focused on Mandarin-speaking children with bimodal stimulation. One study considered both populations. One study was identified to be relevant to bilateral CI pediatric recipients.

## Review

### Auditory and Speech Perception Measures

When selecting outcome measures for children, it is important to take into account a variety of factors, including chronological age, developmental status, vocabulary and language competency. Age-appropriate auditory and speech perception outcomes may include self-report questionnaires and behavioral measures (see Tables 1, 2 for a summary). Four parental questionnaires were identified, including the Meaningful Auditory Integration Scale (MAIS)/Infant-Toddler Meaningful Auditory Integration Scale (ITMAIS; Zheng et al., 2009c), the LittleEARS^®^ Auditory Questionnaire (LEAQ; Wang et al., 2013), the Categories of Auditory Performance Questionnaire (CAPQ; Wang et al., 2020), and the Parent’s Evaluation of Aural/Oral of Children (PEACH) rating scale (Zhang et al., 2021). These parental questionnaires could be utilized to evaluate preverbal, early auditory, and speech perception in children up to 6 years of age, when children have limited language skills and speech perception measures are difficult to administer. The IT-MAIS/MAIS, LEAQ and CAPQ have been used extensively in research institutes and clinics in China. Since the PEACH rating scale is newly developed, few studies have employed this measure.

**TABLE 1:** Parental questionnaires.

Studies	Test name	Test materials	Content	Reliability and validity reported	Target age (years)
Zheng et al., 2009c	The IT-MAIS The MAIS	10 items in 3 categories on a 5-point scale	Self-report about device/vocal behavior and device use; spontaneous detection of and response to sounds; spontaneous and meaningful recognition and discrimination of sounds	Internal consistency (Cronbach’s α = 0.96; Guttmann’s split-half coefficient = 0.96), item reliability (Pearson’s *r* with other items and overall scores = 0.70–0.89)	IT-MAIS: 2–3 MAIS: ≥ 3
Wang et al., 2013	The LEAQ	35 yes-or-no questions	Observed receptive, semantic and early expressive language skills, such as response to a familiar voice or whether simple questions can be understood	Predictability (Guttman’s lambda = 0.882), internal consistency (Cronbach’s α = 0.945; Spearman–Brown split-half coefficient = 0.914), validity (Pearson’s *r* between age and total scores = 0.841)	<2
Wang et al., 2020	The CAPQ	10 categories from Level 0 to Level 9	Hierarchical categories on children’s auditory abilities, ranging from Level 0 indicating no awareness of environmental sounds to Level 9 indicating the ability to use the phone with unknown speakers in unpredictable context.	Test-retest reliability (Spearman’s *r* coefficient = 0.981), inter-rater reliability (Pearson’s *r* = 0.983) and criterion validity (Pearson’s *r* with the LEAQ: *r* = 0.721)	0–6 (tested^1^)
Zhang et al., 2021	The PEACH rating scale	12 items on a 5-point scale	Aural/oral behaviors in real-world quiet and noisy listening conditions, such as being able to follow simple instructions in a quiet or noisy situation	Test–retest reliability (Cronbach’s α = 0.98; correlation coefficient^2^: *r* = 0.96) and validity (correlation coefficient^2^ with the PCDI: *r* = 0.42)	<4

*The CAPQ, The Categories of Auditory Performance questionnaire; The IT-MAIS, Infant-Toddler Meaningful Auditory Integration Scale; The LEAQ, The LittleEARS^®^ Auditory Questionnaire; The MAIS, The Meaningful Auditory Integration Scale; The PCDI, Putonghua Communicative Development Inventory; The PEACH rating scale, The Parent’s Evaluation of Aural/Oral of Children Rating Scale.*

*^1^The study did not indicate the target age and thus the age range of participants in the study is reported.*

*^2^The study did not indicate the type of correlation analysis.*

**TABLE 2:** Speech perception tests.

Studies	Test name	Type of test materials	Paradigm	Test in quiet and/or noise	Homogeneity	Target age (years)	Reliability and validity reported
Zheng et al., 2009a	The MESP test	A hierarchically structured test with six categories in speech sound and pattern, spondee, vowel, consonant and tone.	Closed-set recognition 12-AFC or other depending on the category	Quiet	Not reported	≥2	Not reported
Zheng et al., 2009b	The MPSI test	Sentences with 6–7 characters	Closed-set recognition 6-AFC	Quiet and/or in competing sentence at fixed SNRs from +10 to –10 dB	Not reported	3–6 (tested^1^)	Not reported
Yuen et al., 2009b	The MAPPID-N	Disyllables and lexical tones	Closed-set recognition 8-AFC for Disyllable test 4-AFC for Lexical tone test	Speech spectrum shaped noise	Across items	4–9 (tested^1^)	Not reported
Xi et al., 2009	MBKB-SIN	Sentences with 6–8 characters	Open-set recognition	Quiet and/or in four-talker babble noise	Across lists	4.5–6 (tested^1^)	Test–retest reliability (critical difference: 24.6%)
Liu C. et al., 2011	The LNT	Easy and hard Monosyllables and disyllables	Open-set recognition	Quiet	Across lists	4–7 (tested^1^)	Inter-rater reliability (consistency between two raters: 92.5–95%)
Zhu et al., 2014	The MTIT	Lexical tones	Closed-set recognition 4-AFC with 1 target word, 1 tone contrast and 2 unrelated distracters	Quiet and/or in speech spectrum shaped noise at fixed SNRs	Not reported	≥7	Internal consistency (Cronbach’s α = 0.66-0.76), Test–retest reliability (intra-class correlation = 0.65–0.71), criterion validity [correlated with MPSI in quiet (Kendall’s tau = 0.33) and in noise (Spearman’s *r* = 0.71) (Zhu et al., 2016)]
Chen and Wong, 2020	The MHINT-C	Sentences with 10 characters	Open-set SRT	Quiet and/or in Steady-state-speech-spectrum-shaped noise at adaptive SNRs	Across lists	6–17 (tested^1^)	Inter-list reliability (confidence intervals: ± 2.8 dB), response variability (1.90-2.0 dB)

*AFC, alternative forced choice; MBKB-SIN, Mandarin Bench-Kowal-Bamford sentence in Noise Test; The LNT, the Standard-Chinese version of the Lexical Neighborhood Test; The MAPPID-N, The computerized Mandarin Pediatric Lexical Tone and Disyllabic-word Picture Identification Test in Noise; The MESP test, the Mandarin Early Speech Perception test; The MHINT-C, The Mandarin version of the Hearing in Noise Test for Children; The MPSI, The Mandarin Pediatric Speech Intelligibility test; The MTIT, The new Mandarin Tone Identification Test; SNR, Signal-to-noise ratio; SRT, Speech Recognition Threshold.*

*^1^The study did not indicate the target age and thus the age range of participants in the study is reported.*

**FIGURE 2:**
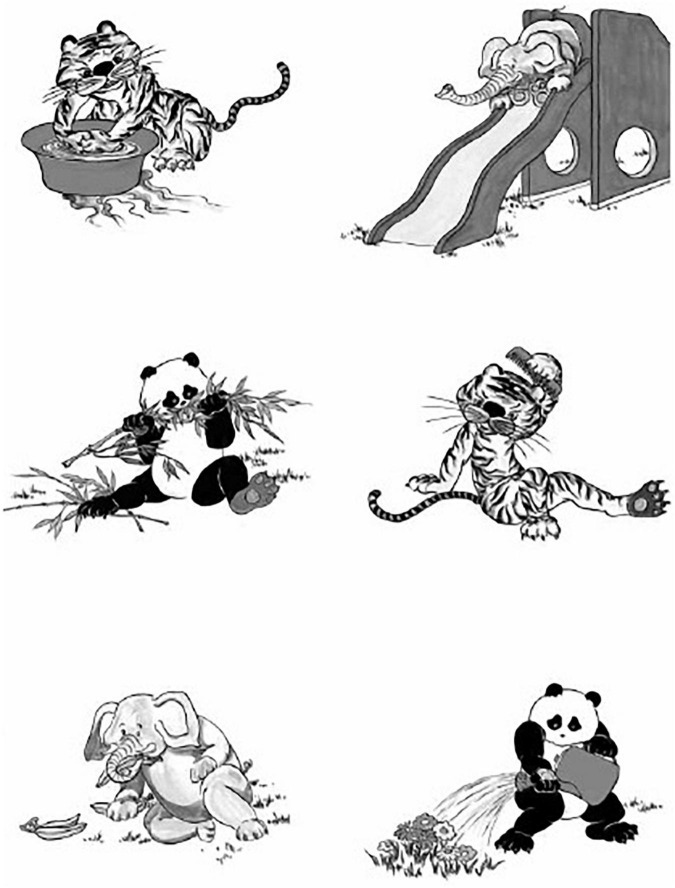
A picture plate for the MPSI test from Zheng et al. (2009b).

Multiple measures were developed to evaluate the perception of phonemes, lexical tones, mono- and multi-syllables, and sentences in quiet and/or in noise. Considering the developmental capabilities of young children, the majority of tests are administered in a closed-set paradigm, in which children point to objects or select answers from a picture panel (Figure 2). Open-set tests are used for older children by requesting them to verbally repeat words they heard. Although materials developed by Sun et al. (1993) and Chen X. et al. (2007) are popular, they were mainly developed for use in rehabilitation. Thus, they are not presented in the summary table. Phoneme perception could be evaluated using the vowel (category 4) and consonant (category 5) sub-tests of the Mandarin Early Speech Perception (MESP) test (Zheng et al., 2009a). Lexical tone perception is evaluated using category 6 of the MESP test, the Mandarin Tone Identification Test (MTIT; Zhu et al., 2014), and tone test of the computerized Mandarin Pediatric Lexical Tone and Disyllabic-word Picture Identification Test in Noise (MAPPID-N; Yuen et al., 2009b). Syllable perception could be measured using the spondee perception (category 2) sub-test of the MESP test, disyllables test of the MAPPID-N, and Standard-Chinese version of the Lexical Neighborhood Test (LNT; Liu C. et al., 2011). Sentence recognition is evaluated using the Mandarin Pediatric Speech Intelligibility (MPSI) test (Zheng et al., 2009b), the Mandarin Bench–Kowal–Bamford sentences in noise test (MBKB-SIN; Xi et al., 2009), and the Mandarin version of the Hearing in Noise Test for Children (MHINT-C; Chen and Wong, 2020).

Accuracy of tests depends on the reliability and validity of speech outcome measures. Reliability of a speech test refers to how consistent it measures listeners’ speech perception ability. The consistency across time, raters, and measurement itself are recognized as test–retest reliability, inter-rater reliability, and internal consistency, respectively. Validity refers to how accurate a speech test truly measures the listener’s speech perception ability. These types of validity were commonly evaluated. Construct validity refers to the adherence of speech audiometry to existing theory or knowledge of speech perception. Content validity refers to the extent to which speech audiometry measures all aspects of speech perception. Criterion validity reflects how comparable the measure is to other valid speech audiometry.

All four self-report questionnaires considered one or two reliability assessments in the development process, in the form of internal consistency (Zheng et al., 2009c; Wang et al., 2013), test–retest reliability (Wang et al., 2020; Zhang et al., 2021), and inter-rater reliability (Wang et al., 2020). Criterion validity was assessed for the CAPQ and the PEACH rating scale.

Among the behavioral measures, item or list equivalence was mostly established by measuring psychometric functions and adjusting the intensity of corresponding mean recognition scores and/or mean slope at 50%. While inter-list or test-retest reliability was assessed for some measures (i.e., the MBKB-SIN and the MHINT-C), and certain criteria were applied in constructing the items (e.g., vocabulary, familiarity, phonetically balancing for phoneme distribution and lexical tones), other types of validity was seldom reported. In fact, normative data were mostly collected on NH listeners and researchers rarely validated these measures on listeners with HL or CI, whose performance varies greatly within the group and the error patterns in performance may differ from NH listeners (Li et al., 2016).

### Outcomes With Unilateral Cochlear Implantation

The majority of CI users in mainland China are using unilateral implants. There are 31 studies examining outcomes from unilateral CI; among them, 16 are cross-sectional and 15 are longitudinal. Demographic factors were evaluated in both types of studies in order to explain performance variability. As all but one study on lexical tone perception have been reviewed by Chen and Wong (2017), they are not reviewed here. For this review, we focused on longitudinal studies on unilateral CI to synthesize evidence. A summary of results from cross-sectional studies can be found in Supplementary Material.

#### Longitudinal Studies on the Development of Auditory and Speech Perception

The 15 longitudinal studies focused on the developmental trajectory of children with congenital HL and used unilateral CI for not more than 7 years (see Table 3 for a summary). The age of implantation ranged from an average of 1.58–8.86 years across studies. Auditory behavior, perception of phonemes, syllables and sentences in quiet and in noise were evaluated, demonstrating continuous improvement in early auditory behavior and early speech perception after the device activation, up to 5 years post implantation.

**TABLE 3:** Longitudinal studies on speech perception with unilateral CI.

Studies	Participant characteristics	Outcome measures^1^	Overall results^1^
Chen X. et al., 2010 (*N* = 259)	AAI (years): *M* = 1.8, *R* = 0.7–3.0 Tested at baseline, 1-, 2-, 3-, 6-, 12-months post CI	The IT-MAIS	Early auditory skills improved significantly over time.
Zheng et al., 2011 (*N* = 39)	AAI (years): 1–2 (*n* = 4), 2–3 (*n* = 12), 3–4 (*n* = 12), 4–6 (*n* = 11) Tested at baseline, 3-, 6-, and 12-months post CI	The IT-MAIS The MESP test The MPSI test	Early pre-lingual auditory development and early speech perception were comparable to English-speaking children.
Li Y. et al., 2015 (*N* = 22)	AAI (years): *M* = 2.9, *R* = 1.1-5.7 Tested at baseline, 1-, 3-, 6-, 9-, 12-, 24-, and 36-months post CI	The MESP test	Speech performance through the first 3 years of implant use, with the median categories of MESP increased from a score of 0.23 indicating barely any speech detection at baseline to 5.57 suggesting phoneme, tone and word recognition 3 years later.
Liu et al., 2015a (*N* = 33)	AAI (years): *M* = 2.02, *SD* = 0.89, *R* = 0.5–3.83 Tested at baseline, 1-, 3-, 6-, 9-, 12-, 18-, and 24-months post CI	The LEAQ	Auditory preverbal skills improved significantly post CI in the first 2 years of use.
Liu et al., 2015b (*N* = 105)	AAI (years): *M* = 3.1, *SD* = 2.3, *R* = 0.9-15.5 Tested at 6-, 12-, 24-, 36-, 48-, 60-, 72-, and 84-months post CI	The LNT	Spoken word recognition improved significantly over time. The fastest improvement occurred in the first 36 months, after which it slowed down and peaked at 72 months post CI (81.7%).
Chen Y. et al., 2016 (N = 80)	AAI (years): M = 2.61, SD = 1.04, R = 0.93-5.00 Tested at baseline, 3-, 6-, and 12-months post CI	The IT-MAIS/ MAIS The MESP test The MPSI test	Significant progress in prelingual auditory, word and sentence recognition were observed during the first year of CI use. Mandarin-speaking children with CIs attained early speech perception results comparable to those of their English-speaking counterparts.
Guo et al., 2016 (*N* = 23)	AAI (years): *M* = 3.0, *R* = 1.08-5.67 Tested at 1-, 2-, 3-, and 4-years post CI	The MESP test	The proportion of participants having reached higher categories increased significantly during the 4 years post CI. The percentage of participants passing category 6 (tone perception) of the MESP increased from 9% at 1st year to 91% at 4th year post CI.
Liu et al., 2016 (*N* = 213)	AAI (years): The mean ranged from 2.49 to 3.15 in groups of different etiology. Tested at baseline and 1-year post CI	Mono-, di-syllable and sentence recognition	Significant improvement in recognition of monosyllabic, disyllabic words and sentences at 1 year post CI.
Li G. et al., 2017 (*N* = 143)	AAI (years): 1–2 (*n* = 34), 2–3 (*n* = 72), 3–4 (*n* = 37) Test at before, 2-, 6-, 12-, 24-, 36-, 48-months post CI	Tone perception subset in the MESP test	Mean identification score increased from approximately 68% to 79% by 4 years post CI.
Lu and Qin, 2018 (*N* = 132)	AAI (years): *M* = 3.4 *SD* = 1.35 Tested at baseline, 3-, 6-, 9-, 12-, 18-, and 24-months post CI	The IT-MAIS The MESP test	Significant improvements in early auditory and speech development that follow the normative developmental trajectories. However, there was still a gap (10–15%) compared with normative values.
Liu S. et al., 2019 (*N* = 98)	AAI (years): *M* = 8.86, *SD* = 3.66, *R* = 1.0–16.0 Tested at baseline, 3-, 6-, and 12-months post CI	Speech perception^2^ The MAIS The CAPQ	The scores of all measures significantly improved at 1 year post CI.
Lyu et al., 2019 (*N* = 278)	AAI (years): *M* = 1.58, *R* = 0.5–3.0 Tested at baseline, 1-, 3-, 6-, 12-, 18-, 24-, 36-, 48-, 60-months post CI	The CAPQ	Scores improved during the 5 years post CI, although speech development lagged behind that of hearing.
Fan et al., 2020 (*N* = 52)	AAI (years): Median = 1.25, *R* = 0.83–5.66 Tested at baseline, 3-, 6-, 9-, 12-, 15-, 18-, 21- and 24-months post CI	Closed monosyllables and disyllables recognition The CAPQ	Auditory and speech perception improved significantly over the 24 months post CI period.
Jiang et al., 2020 (*N* = 100)	AAI (years): Median = 4.0, *R* = 3.0–7.0 Tested at 1 months, 1-, 2-, and 3-years post CI	The CAPQ	Significant improvements in the CAPQ scores at 3 years post CI. 60% of children reached Level 7 indicating children were able to use the telephone with a familiar talker at 3 years post CI.
Li G. et al., 2020 (*N* = 24)	Age at Switch-on: *M* = 2.1, *SD* = 0.47, *R* = 1.2–2.8 Tested at baseline, 3-, 6-, and 12-months post CI	The IT-MAIS	Children below 3 years of age had similar trajectories in early auditory developments to NH children.

*AAI, Age at Implant; CI, Cochlear Implantation; M, Mean; N, The Number of Participants; R, Range; SD, Standard Deviation.*

*^1^Only outcome measures and results related to speech perception were reported.*

*^2^Speech perception here referred to Chinese auditory perception and open-set speech perception.*

Data showed no or very low level of auditory skills pre-implant. After 6 months of CI use, these children could achieve a score of about 50∼60% on the IT-MAIS/MAIS (Chen Y. et al., 2016; Lu and Qin, 2018; Li G. et al., 2020) and reach category 3 (i.e., recognizes environmental sounds) on the CAPQ (Lyu et al., 2019; Chen Y. et al., 2020; Jiang et al., 2020). About 40∼88% of children reached category 2 (i.e., speech pattern perception) or higher on the MESP test (Zheng et al., 2011; Chen Y. et al., 2016; Guo et al., 2016; Lu and Qin, 2018). Approximately 7.9∼20.6% of children could obtain a score of 25∼42% for close-set sentence perception on the MPSI in quiet (Zheng et al., 2011; Chen Y. et al., 2016) and participants in Liu et al. (2015b) achieved an average score of 30.9% for mono- and disyllable recognition on the LNT. These results suggest that at 6 months post CI, children begin to develop closed-set word recognition and sentence recognition in quiet, as well as open-set word recognition in quiet. At 12 months post CI, children could obtain scores of about 70∼80% on the IT-MAIS/MAIS (Chen Y. et al., 2016, 2020; Lu and Qin, 2018; Li G. et al., 2020) and reach category 4 (i.e., discriminates at least two speech sounds) on the CAPQ (Lyu et al., 2019; Chen Y. et al., 2020; Jiang et al., 2020). More than half of the children could achieve category 4 (i.e., vowel perception) and category 5 (i.e., consonant perception) on the MESP test (Zheng et al., 2011; Chen Y. et al., 2016; Guo et al., 2016; Lu and Qin, 2018). About 33.9–56.7% of the children could achieve a mean score of 60–70% for closed-set sentence recognition on the MPSI test in quiet and a similar proportion of children could obtain a mean score of 46–59% on the MPSI test in noise (Zheng et al., 2011; Chen Y. et al., 2016). The mean recognition scores in monosyllables, disyllables and sentences increased significantly to 78.60, 88.57, and 89.79% respectively at 1-year post-operatively from a baseline of 13∼42% pre-implant (Liu et al., 2016). These results suggest that at one-year post-operation, children with unilateral CI could demonstrate a good ability to identify closed-set words and sentences in quiet; and some children could develop the ability to identify sentences in noise. Greatest improvement in open-set word recognition occurs between 1 and 3 years after surgery and then reaches a plateau at 48 months (Liu et al., 2015b). All children could develop tone recognition ability (category 6 of the MESP test) after 4 to 5 years of CI use, and 60–80% of children showed lexical tone recognition significantly higher than the chance level (Li G. et al., 2017).

From these findings, a clear trajectory of development on auditory behaviors and closed-set phoneme recognition is observed. However, there are few reports on sentence and open-set words recognition. Many studies (6 out of 15) reported findings from 1-year post-implantation, thus allowing insufficient time to develop mastery of complex grammatical skills and lexicons to be assessed in open-set word recognition tasks or sentence tests, which is more demanding than phoneme and closed-set word recognition. Longer follow-up period is necessary in order to observe the performance trajectory over time. In addition, considering various tests were used on participants with different demographic factors such as age at implantation (AAI) [e.g., mean AAI was 1.58 years in Lyu et al. (2019) and 8.86 years in Liu S. et al. (2019)], whether HAs were trialed pre-implant, and whether speech therapy was provided post-implant, performance varied across participants and studies, decreasing the ability of this review in generalizing findings.

#### Important Factors That Affect Speech Perception

A summary of frequently examined factors among studies can be found in Table 4. Details about less-frequently examined factors (≤3 studies) are presented in Supplementary Materials. AAI, duration of CI use (DCI), whether there was a pre-CI hearing aid trial (HAT), and caregiver education level (CEL) are discussed below and more than half of analyses that investigated these variables show that they significantly impacted speech perception outcomes.

**TABLE 4:** Frequently examined factors that affect speech perception of children with unilateral CI.

Studies	Participant characteristics	Outcome measures^1^	Analysis method	AAT	AAI	CEL	DCI	HAT	PHL
Han et al., 2009 (*N* = 20)	AAI: *M* = 5.2, *SD* = 3.8, *R* = 1.3–13.5 years	Lexical tone recognition in quiet	Least-squared linear fit	√*	√*	–	√	–	–
Chen X. et al., 2010 (*N* = 259)	AAI: M = 1.8, *R* = 0.7–3.0 years Tested at baseline, 1-, 2-, 3-, 6-, 12-months post CI	The IT-MAIS	ANOVA and *t* test	–	√	–	√*	√*	–
Zhu et al., 2011 (*N* = 37)	AAI: *M* = 4.2, *R* = 1.2–17.5 years (Group 1: Congenitally deafened children)	Open-set disyllables recognition Sentences recognition	Multiple linear regression	√	√*	–	–	–	–
				√*	√*	–	–	–	–
Zheng et al., 2010 (*N* = 25)	AAI: *M* = 3.39, *R* = 1.5–9.1 years	The MESP test	DNR	–	√	–	√	–	–
Zheng et al., 2011 (*N* = 39)	AAI: 1–2 years (*n* = 4), 2–3 years (*n* = 12), 3–4 years (*n* = 12), 4–6 years (*n* = 11) Tested at baseline, 3-, 6-, and 12-months post CI	The IT-MAIS	Pearson’s correlation and χ^2^ test of independence	–	√*	–	–	√*	–
		The MESP and MPSI test		–	–	–	–	√*	–
Liu H. et al., 2013 (*N* = 230)	AAI: *M* = 3.9, *SD* = 3.0, *R* = 0.9-16.0 years	Open-set word recognition	Stepwise multiple regression	–	√*	–	√*	–	–
Liu Q. et al., 2013 (*N* = 41)	AAI: *M* = 2.0, *SD* = 0.74, *R* = 0.83–4.17 years	Mandarin consonant contrast perception	Linear regression	√	√*	–	√	–	√
Zhou et al., 2013 (*N* = 110)	AAI: *M* = 3.96, *SD* = 2.70, *R* = 1.11–12.95 years	Lexical tone recognition in quiet	Step-wise linear regression	√	√	-	√*	-	-
Li A. et al., 2014 (*N* = 20)	AAI: *M* = 4.1, *R* = 2.0–6.7 years	Lexical tone recognition in quiet	Linear regression	√*	√*	–	√	–	√
Chen Y. et al., 2014 (*N* = 96)	AAI: *M* = 2.72, *SD* = 1.03, *R* = 0.69–5.00 years	Lexical tone perception in quiet	Step-wise multiple linear regression	–	√	√	√*	√	√
		Sentence perception in quiet		–	√	√*	√*	√	√
		Sentence perception in noise		–	√	√*	√*	√*	√*
Chen Y. et al., 2015^2^ (*N* = 115)	AAI: *M* = 2.67, *SD* = 1.08, *R* = 0.69–5.00 years	Overall speech perception^3^	Structural equation modeling	–	√*	√*	–	√*	√
Li Y. et al., 2015 (*N* = 22)	AAI: *M* = 2.9, *R* = 1.1–5.7 years Tested at baseline and 1-, 3-, 6-, 9-, 12-, 24-, and 36-months post CI	The MESP test	Repeated-measure ANOVA	–	√*	–	√*	–	–
Liu et al., 2015a^4^ (*N* = 33)	AAI: *M* = 2.02, *SD* = 0.89, *R* = 0.5–3.83 years Tested at baseline, 1-, 3-, 6-, 9-, 12-, 18-, and 24-months post CI	The LEAQ	ANOVA	–	√*	√*	√*	–	–
Liu et al., 2015b (*N* = 105)	AAI: *M* = 3.1, *SD* = 2.3, *R* = 0.9–15.5 years Tested at 6-, 12-, 24-, 36-, 48-, 60-, 72-, 84-months post CI	Open-set word recognition	ANOVA	–	√*	–	√*	–	–
Tao et al., 2015 (*N* = 21)	AAI: *M* = 4.3, *R* = 2–12 years (Prelingual group)	Lexical tone perception in quiet	Linear regression	–	–	–	√	–	–
Chen Y. et al., 2016 (*N* = 80)	AAI: *M* = 2.61 *SD* = 1.04, *R* = 0.93–5.00 years Tested at baseline, 3-, 6-, and 12-months post CI	Overall speech perception^3^	Hierarchical linear modeling	–	√*	√*	√*	√	√*
Mao and Xu, 2017^5^ (*N* = 66)	AAI: *M* = 2.97, *SD* = 3.05, *R* = 0.6–16.50 years	Lexical tone recognition in quiet and in noise	Linear correlation	√	√*	–	√	–	–
Li G. et al., 2017 (*N* = 143)	AAI: 1–2 years: *n* = 34; 2-3 years: *n* = 72; 3–4 years: *n* = 37 Test at baseline, 2-, 6-, 12-, 24-, 36-, and 48-months post CI	Tone perception subset in the MESP test	Two-sample *t* test	–	√	–	√*	–	–
Lu and Qin, 2018^6^ (*N* = 132)	AAI: *M* = 3.4 *SD* = 1.35 years Tested at baseline, 3-, 6-, 9-, 12-, 18-, and 24-months post CI	The IT-MAIS	Multiple linear and logistic regression	–	√*	–	√*	√*	√*
		The MESP test		–	√*	–	√*	√	√*
Liu S. et al., 2019 (*N* = 98)	AAI: *M* = 8.86, *SD* = 3.66, *R* = 1.0–16.0 years Tested at baseline, 3-, 6-, and 12-months post CI	The MAIS, the CAPQ and speech perception^7^	ANOVA	–	√*	–	√*	–	–
Lyu et al., 2019 (*N* = 278)	AAI: *M* = 1.58, *R* = 0.5–3.0 years Tested at baseline, 1-, 3-, 6-, 12-, 18-, 24-, 36-, 48-, and 60-months post CI	The CAPQ	*t* test and linear regression	–	√	–	√*	–	–
Fan et al., 2020^8^ (*N* = 52)	AAI: Median = 1.25, *R* = 0.83–5.66 years Tested at baseline, 3-, 6-, 9-, 12-, 15-, 18-, 21- and 24-months post CI	Closed-set monosyllables Closed-set disyllables The CAPQ	Generalized estimating equation	–	√*	√*	√*	√*	√
Jiang et al., 2020 (*N* = 100)	AAI: Median = 4.0, *R* = 3.0–7.0 years Tested at 1 months, 1-, 2-, and 3-years post CI	The CAPQ	Mann-Whitney test	–	√*	–	√*	√*	–
Li G. et al., 2020 (*N* = 24)	Age at Switch-on: *M* = 2.1, *SD* = 0.47, *R* = 1.2–2.8 years Tested at baseline, 3-, 6-, and 12-months post CI	The IT-MAIS	Mann-Whitney test	–	√	–	√*	–	–

*AAI, Age at Implant; AAT, Age at Testing; ANOVA, Analysis of Variance; CEL, Caregiver’s Educational Level; CI, Cochlear Implantation; DCI, Duration of CI use; DNR, Did not report the statistical test used; HAT, Hearing Aid Trial; M, Mean; N, The Number of Participants; PHL, Pre-implant Hearing Level; R, Range; SD, Standard Deviation.*

‘√*’ shows that this study examined the corresponding factor and found a significant correlation. ‘**√**’ shows that this study examined the corresponding factor but no significant relationship was found. ‘**–**’ shows that this study did not examine the corresponding factor.

*^1^Only outcome measures and results related to speech perception were reported.*

*^2^In Chen Y. et al. (2015), 5 children used a HA in the non-implanted ear and were tested with both CI and HA on.*

*^3^Overall speech perception referred to a single composite score was generated by combining results from MAIS, the MESP test and the MPSI test using the principal component analysis.*

*^4^In Liu et al. (2015a), 1 child received bilateral CIs.*

*^5^In Mao and Xu (2017), the significance for AAI became non-significant after correction for multiple comparisons.*

*^6^In Lu and Qin (2018), only results at 1 year post CI were presented due to space limitation.*

*^7^Speech perception here referred to Chinese auditory perception and open-set speech perception.*

*^8^6. In Fan et al. (2020), 9 (18.4%) children used bilateral CIs and 11 (22.4%) children used CI + HA.*

Early AAI, similar to studies on an English-speaking population (see a review from, for example, Bruijnzeel et al., 2016; Sharma et al., 2020), is associated with enhanced speech perception in children. Seven longitudinal studies reported that early implantation contributed positively to prelingual auditory skills and early speech perception evaluated on the IT-MAIS/MAIS, the LEAQ, the MESP test, the MPSI test, and the LNT (Liu et al., 2015a; Chen Y. et al., 2016; Lu and Qin, 2018; Liu S. et al., 2019; Lyu et al., 2019; Fan et al., 2020; Jiang et al., 2020).

Longer DCI significantly contributes to better auditory skills and speech perception in all longitudinal studies. Open-set word recognition and sentence recognition were significantly correlated with longer DCI in cross-sectional studies, as reported in the previous review (Liu H. et al., 2013; Chen Y. et al., 2014). Lexical tone recognition, however, was not correlated with DCI in 4 out of 6 cross-sectional studies that conducted such analyses (Han et al., 2009; Li A. et al., 2014; Tao et al., 2015; Mao and Xu, 2017). Participants in studies that demonstrated a lack of effects of DCI used their devices longer (*M* = 2.36–6.50 years) than those in the two studies (Mean DCI = 1.27–1.64) that found significant correlations (Zhou et al., 2013; Chen Y. et al., 2014). The only study assessing the effect of DCI on Mandarin consonant contrast perception also showed no significant correlation (Liu Q. et al., 2013).

Having undergone a HAT before CI is a factor that positively influences the auditory development and speech perception. All longitudinal studies that assessed the relationship between receiving HAT prior to implantation and auditory scores showed significant effects (Chen X. et al., 2010; Lu and Qin, 2018; Fan et al., 2020; Jiang et al., 2020). However, mixed findings were reported among studies on early speech perception, with Zheng et al. (2011) and Fan et al. (2020) reporting significant effects of HAT on closed monosyllable and disyllable recognition and the MESP scores, but Chen Y. et al. (2016) and Lu and Qin (2018) did not observe such correlations with early speech perception. In a cross-sectional study, having undergone HAT was associated with better sentence recognition in noise, but not with sentence and tone recognition in quiet (Chen Y. et al., 2016).

Better caregiver’s education contributed positively to preverbal auditory skills (Liu et al., 2015a), overall early speech perception (Chen Y. et al., 2015, 2016; Fan et al., 2020) and sentence perception in quiet and in noise (Chen Y. et al., 2014). However, Chen Y. et al. (2014) did not find parental education relate to lexical tone recognition in quiet. Such variables are specified as parents’ education levels in Liu et al. (2015a) and maternal education level in Chen Y. et al. (2020, 2014, 2015) and therefore cannot be directly compared. Interestingly, Fan et al. (2020) found that children who were cared for by their mothers exhibited better closed monosyllable recognition rates, than those who were cared for by their grandparents.

### Outcomes With Bimodal Stimulation

With improving socioeconomics and greater recognition of the importance of binaural hearing, bimodal stimulation is gradually becoming a key focus of researchers, clinicians and parents in mainland China. Bimodal stimulation refers to the combination of a CI in the implanted ear and a HA in the non-implanted ear. Adding a contralateral HA allows unilateral CI users to exploit the residual hearing in the non-implanted ear, reducing auditory deprivation and enabling binaural hearing (Hurley, 1999; Polonenko et al., 2018). Bimodal benefits in sound localization, music perception, and speech perception for non-tonal language speakers such as English have been established by a huge body of evidence (Ching et al., 2007). For example, speech perception in noise could be enhanced through binaural summation, head shadow effect, and squelch effect (Lotfi et al., 2019).

The contribution of the F0 in the low-frequency range is important for Mandarin perception. Thus, a contralateral HA that delivers amplification in this frequency range may produce unique bimodal benefits for the Mandarin-speaking population. One longitudinal and five cross-sectional studies were identified through the literature search, comparing speech perception with bimodal stimulation and CI only condition (see Table 5 for a summary).

**TABLE 5:** Speech perception of children with bimodal stimulation.

Studies	Participant characteristics	Device settings	Outcome measures^1^	Overall results
Yuen et al., 2009a (*N* = 15)	Age (years): *M* = 10.2, *R* = 5.1–14.3 DCI (years): *M* = 2.3, *R* = 0.3-6.7	HA fitting was optimization based on the NAL-RP prescription formula	Lexical tones and disyllabic words in quiet and in noise (The MAPPID-N) Test settings: CI-only, CI + HA	Significant bimodal benefits^2^ in lexical tone recognition in quiet and in noise, and disyllabic words in noise when speech and noise both presented from the front.
Cheng et al., 2018 (*N* = 35)	AAI (years): *M* = 2.9, *R* = 0.9–7.0 DCI (years): *M* = 3.5, *R* = 0.6–8.1 DHA (years): *M* = 2.7, *R* = 0.5–9.0	Participants used their clinical settings for CI and HA	Mandarin tone recognition in quiet Vowel recognition in quiet Consonant recognition in quiet Sentence recognition in quiet Test settings: CI-only, CI + HA	Significant bimodal benefits for tone recognition in quiet (Tone 2), but not for vowel, consonant or sentence recognition in quiet.
Liu Y. W. et al., 2019 (*N* = 11)	Age (years): *M* = 8.2, *R* = 6.0–12.5 DCI (years): *M* = 4.5, *R* = 2.0–8.0 DHA (years): *M* = 4.0, *R* = 0.5–8.0	Participants used their clinical settings for CI and HA	Sentence recognition in steady-state noise and in a competing talker Test settings: CI-only, CI + HA	With 2-keywords scoring, no bimodal benefit in steady-state noise and female competing talker. Bimodal stimulation resulted in better scores than the CI-only condition. With 5-keywords scoring, significant bimodal benefits were observed.
Chen Y. et al., 2020 (*N* = 28)	AAI (years): *M* = 1.47, *SD* = 0.57 Tested at first mapping, 0.5-, 1-, 3-, 6-, 12-, 18-, and 24-months after	CI mapping and HA fitting were carried out by experienced clinicians	The IT-MAIS The CAPQ Test settings: CI + HA for the bimodal group	The bimodal group demonstrated significantly higher scores at baseline, 3-, and 6-months on the IT-MAIS and from 3- to 24 months on the CAPQ compared to the unilateral CI group.
Zhang et al., 2020a (*N* = 14)	AAI (years): *M* = 1.96, *R* = 0.9-3.3 DCI (years): *M* = 3.59, *R* = 2.3-5.2 Bimodal duration (years): *M* = 3.23, *R* = 1.7–5.0	Participants used their daily settings for CI and HA	Lexical tone recognition in quiet and in speech spectrum-shaped noise at + 5 dB Test settings: CI-only, CI + HA	Significant improvement was seen in the CI + HA condition over the CI-only condition for lexical tone recognition in noise.
Zhang et al., 2020b (*N* = 16)	AAI (years): *M* = 1.91, *R* = 0.9–3.3 DCI (years): *M* = 3.45, *R* = 2.1–5.1 DHA (years): *M* = 3.45, *R* = 1.7–5.3	Not mentioned	An identification task with a set of synthetic tone-pair continuum (T1-T2) A discrimination task with same stimuli Test settings: CI-only, CI + HA	Significant bimodal benefits in lexical tone categorization were found over the CI-only condition.

*AAI, Age at implant; CI, Cochlear Implantation; DCI, Duration of CI use; DHA, Duration of hearing aid use; HA, Hearing Aid; M, Mean; N, The number of participants; R, Range; SD, Standard deviation; T, Tone.*

*^1^ Only outcome measures and results related to speech perception are reported.*

*^2^Bimodal benefits are measured as a comparison between CI + HA condition over CI-only condition for all studies except Chen Y. et al. (2020) where the comparison was made with a group of participants using unilateral CI.*

Chen Y. et al. (2020) was the only study that retrospectively compared the auditory skills of children with unilateral CI and bimodal stimulation during the 24 months post CI. The AAI in the bimodal group and unilateral CI group was on average of 1.47 and 1.58 years respectively. The bimodal group had better averaged scores compared with the unilateral CI group on the IT-MAIS and CAPQ obtained during follow-up period. The bimodal group obtained nearly full scores on the IT-MAIS faster than the unilateral CI group (18 months vs. 24 months post-implantation). Also, they outperformed the unilateral CI group from 3-months post CI on the CAPQ.

Four out of seven studies evaluated lexical tone perception in quiet and/or in noise. Three of these studies focused on bimodal benefits on lexical tone identification (Yuen et al., 2009a; Cheng et al., 2018; Zhang et al., 2020a), specified as the performance differences of bimodal stimulation (i.e., CI + HA) condition over CI-only condition. All studies found significant bimodal benefits in lexical tone recognition in quiet and/or in noise. Although significant improvement in the recognition of Tone 2 in quiet with bimodal stimulation was noted in Cheng et al. (2018), a ceiling effect was evident where listeners performed nearly perfectly regardless of conditions (CI + HA or CI-only). Zhang et al. (2020a) showed bimodal benefits in lexical tone recognition in speech spectrum-shaped noise at +5 dB but not in quiet, whereas Yuen et al. (2009a) also found significant bimodal benefits in lexical tone recognition when speech was presented from the front and noise from the CI side. Zhang et al. (2020b) investigated categorial perception using synthetic tone-pair continuums, showing enhanced categorical perception in Tone 1–2 continuums with bimodal stimulation compared to CI-only condition.

Vowel, consonant, disyllable and sentence recognition was assessed in three studies (Yuen et al., 2009a; Cheng et al., 2018; Liu Y. W. et al., 2019). Yuen et al. (2009a) reported significant benefits in disyllable recognition when speech was presented from the front and noise was presented on the CI side. Vowel, consonant and sentence recognition were measured in quiet and no significant bimodal benefits were found (Cheng et al., 2018). Liu Y. W. et al. (2019) compared speech reception thresholds (SRTs) in different maskers with and without HAs using 2-keywords scoring. While performance in steady-state noise (SSN) and the female competing talker did not differ, SRTs with bimodal listening was worse when competing and target voices were the same, indicating bimodal interference. In the second experiment of this study, using 5-keywords scoring, a significant bimodal benefit in SRTs in the presence of SSN was evident, indicating bimodal benefits in more challenging tasks.

Four out of six studies examined the correlation between demographic factors and speech perception (Yuen et al., 2009a; Cheng et al., 2018; Liu Y. W. et al., 2019; Zhang et al., 2020a). A summary of these studies can be found in Table 6. Effects of hearing thresholds in the non-implanted ear were examined in all four studies. Significant correlations were found between low-frequency hearing thresholds in the non-implanted ear and disyllables recognition in noise (Yuen et al., 2009a), lexical tone recognition in noise (Zhang et al., 2020a); and sentence recognition in quiet (Cheng et al., 2018) and in noise (Liu Y. W. et al., 2019) (please see Table 6). Similar to studies in unilateral CI use, age at testing, AAI and DCI were examined. Cheng et al. (2018) found that AAI significantly correlated with lexical tone and consonant recognition in quiet with bimodal stimulation. Cheng et al. (2018) and Zhang et al. (2020a) both found that DCI significantly correlated with lexical tone recognition in quiet with bimodal stimulation. Duration of deafness was examined in two studies, but only Cheng et al. (2018) found bimodal stimulation significantly correlated with consonant recognition in quiet. Duration of bimodal use was examined in Zhang et al. (2020a) only and the study revealed that bimodal CI was significantly related to lexical tone recognition both in quiet and in noise.

**TABLE 6:** Factors that affect speech perception of children with bimodal stimulation.

Studies	Participant characteristics	Outcome measures	Test setting	Analysis method	AAT	AAI	DCI	DHA	DOB	DOD	PTA
Yuen et al., 2009a^1^ (*N* = 15)	Age (years): *M* = 10.2, *R* = 5.1–14.3 DCI (years): *M* = 2.3, *R* = 0.3–6.7	Lexical tone recognition in quiet and in noise	Bimodal benefits	Pearson’s correlation	–	–	–	–	–	–	√
		Disyllable recognition in noise			–	–	–	–	–	–	√*
Cheng et al., 2018^2^ (*N* = 35)	AAI (years): *M* = 2.9, *R* = 0.9–7.0 DCI (years): *M* = 3.5, *R* = 0.6–8.1 DHA (years): *M* = 2.7, *R* = 0.5–9.0	Tone recognition in quiet Vowel recognition in quiet Consonant recognition in quiet Sentence recognition in quiet	CI + HA	Pearson’s correlation	√	√*	√*	√	–	√	√
					√	√	√	√	–	√	√
					√	√*	√	√	–	√*	√
					√	√	√	√	–	√	√*
Liu Y. W. et al., 2019^3^ (*N* = 11)	Age (years): *M* = 8.2, *R* = 6.0–12.5 DCI (years): *M* = 4.5, *R* = 2.0–8.0 DHA (years): *M* = 4.0, *R* = 0.5–8.0	Sentence recognition in noise	CI + HA	Pearson’s correlation	√	–	√	√	–	√	√*
Zhang et al., 2020a^4^ (*N* = 14)	AAI (years): *M* = 1.96, *R* = 0.9–3.3 DCI (years): *M* = 3.59, *R* = 2.3–5.2 Bimodal duration (years): *M* = 3.23, *R* = 1.7–5.0	Lexical tone recognition in quiet	CI + HA	Multivariate regression	√	√	√*	–	√	–	√
			Bimodal benefits		√	√	√	–	√*	–	√*
		Lexical tone recognition in noise	CI + HA		√	√	√	–	√	–	√
			Bimodal benefits		√	√	√	–	√*	–	√

*AAI, Age at Implant; AAT, Age at Testing; CI, Cochlear Implantation; DCI, Duration of CI use; DHA, Duration of Hearing Aid use; DOB, Duration of Bimodal use; DOD, Duration of Deafness; HA, Hearing aid; M, Mean; N, The number of participants; PTA, Pure Tone Average; R, Range; SD, Standard Deviation.*

‘√*’ Shows that this study examined the corresponding factor and found a significant correlation. ‘**√**’ Shows that this study examined the corresponding factor but no significant relationship was found. ‘**–**’ Shows that this study did not examine the corresponding factor.

*^1^ In Yuen et al. (2009a), PTA referred to aided threshold at 250 and 500 Hz of the non-implanted ear.*

*^2^In Cheng et al. (2018), PTA referred to unaided PTA at 500, 1000, and 2000 Hz.*

*^3^In Liu Y. W. et al. (2019), PTA referred to unaided thresholds in the non-implanted ear at 500 Hz and averaged across all frequencies. The significance was found with bimodal SRTs when the target and masker gender was both male.*

*^4^In Zhang et al. (2020a), PTA referred to three factors including unaided PTA at 125, 250, and 500 Hz, five-frequencies unaided PTA and five-frequencies aided PTA (250–4000 Hz). The significance was only found for the first factor.*

Overall, Mandarin-speaking children with bimodal stimulation seem to outperform unilateral CI users in the development of auditory skills post-implantation, demonstrated as higher scores on the IT-MAIS and CAP during the 24 months post CI (Chen Y. et al., 2020). Better lexical tone recognition in quiet and/or noise is noted with bimodal stimulation, compared to the CI-only condition. Bimodal benefits in speech perception may be related to the task difficulty and more benefits are noted in more challenging situations such as in noise. Apart from lexical tone identification and sentence perception in noise, there is only one study each concerning vowel, consonant and disyllable recognition in quiet, long-term speech perception and the effect of duration of bimodal use. In addition, HA optimization before testing was performed only in Yuen et al. (2009a), which makes comparison with other studies difficult. Therefore, more studies are needed to understand the benefits of bimodal CI compared with unilateral CI.

### Outcomes With Bilateral Cochlear Implantations

Although bilateral CIs have been found to improve speech recognition in noisy conditions over unilateral CI among English-speaking populations (e.g., Asp et al., 2015), reports on bilateral CIs in mainland China did not emerge until 2018. Long et al. (2018) was the only study we identified that investigated the development of early auditory skills in 19 children with simultaneous bilateral CIs. The averaged age at implant was 1.89 years. Participants exhibited continuous improvement in overall LEAQ scores and categorial scores in receptive, semantic auditory behavior and expressive language skills during the 2-year post CI. Children with bilateral CIs obtained significantly higher scores at 1-, 3-, and 6-months post CI than those using unilateral CI (data from Liu et al., 2015a) and the difference nearly disappeared at 24 months post CI. This is possibly due to both groups performing at ceiling. They also found that children whose caregivers have better education and those implanted early tended to exhibit higher LEAQ scores.

## Conclusion

This paper reviewed the literature on speech perception of Mandarin-speaking children with congenital HL and who used CI. Important factors that contribute to individual variations in speech perception outcomes were discussed.

Unilateral CI recipients demonstrated continuous improvements in auditory and speech perception for several years post-activation. Younger AAI and longer DCI contribute to better speech perception. Having undergone a HAT before implantation and having caregivers whose educational level is higher may lead to better performance. While the findings that support the use of CI to improve speech perception continue to grow, much research is needed to validate the use of bimodal and bilateral implantation. Evidence to date, however, revealed bimodal benefits over CI-only conditions in lexical tone recognition and sentence perception in noise. Due to scarcity of research, conclusions on the benefits of bilateral CIs compared to unilateral CI or bimodal CI use cannot be drawn. Therefore, future research on bimodal and bilateral CIs is needed to guide evidence-based clinical practice.

## Author Contributions

QG contributed to the literature review and manuscript drafting. LW contributed to providing review comments. FC contributed to manuscript drafting. All the authors contributed to the article and approved the submitted version.

## Conflict of Interest

The authors declare that the research was conducted in the absence of any commercial or financial relationships that could be construed as a potential conflict of interest.

## Publisher’s Note

All claims expressed in this article are solely those of the authors and do not necessarily represent those of their affiliated organizations, or those of the publisher, the editors and the reviewers. Any product that may be evaluated in this article, or claim that may be made by its manufacturer, is not guaranteed or endorsed by the publisher.
