# Supplementary material for: A Review of Speech Perception of Mandarin-Speaking Children With Cochlear Implantation
Source: Front Neurosci. 2021 Dec 14;15:773694. doi: 10.3389/fnins.2021.773694 (PMC8712552; doi:10.3389/fnins.2021.773694)
Supplement: Supplementary file 1 [file Table_1.docx]

**Supplementary Table 1. Cross-sectional studies on speech perception with unilateral CI**

| Studies | Participants Characteristics | Outcome Measures^1^ | Overall Results^1^ |
| --- | --- | --- | --- |
| Zheng et al, 2011 (N=25) | AAI: M=3.39, R=1.5-9.1years DCI: M=2.89, R=0.9-7.8 years | The MESP test | Among children who reached spondee perception, the proportion of children who could be tested by consonant and tone perception were 36% and 23%, respectively. |
| Zhu et al, 2011 (N=37) | AAI: M=4.2, R=1.2-17.5 years (Group1: Congenitally deafened children) | Open-set disyllables recognition  Sentences recognition | Participants had 82.3% correct for disyllables and 82.8% correct for sentence recognition. |
| Liu, H. et al, 2013  (N=230) | AAI: M=3.9, SD=3.0 R=0.9-16.0 years | Open-set word recognition (The LNT) | Their mean scores for the disyllabic “easy” list, disyllabic “hard” list, monosyllabic “easy” list, and monosyllabic “hard” list were 65.0%, 51.3%, 58.9%, and 46.2%, respectively. |
| Liu, Q. et al, 2013  (N=41) | AAI: M=2.0, SD=0.74, R=0.83-4.17 years | Mandarin consonant phonetic contrast perception | Participants performed from 53.0% to 98.9% correct with a mean of 86.2%. |
| Chen et al, 2014  (N=96) | AAI: M=2.72, SD=1.03, R=0.69-5.00 years | Sentence perception in quiet and in competing speech (The MPSI test) | As the SNR became more difficult, less proportion of children scored significantly above the chance level. |
| Su et al, 2016 (N=11) | AAI: M=4.5 years  DCI: M=5.2 years | Sentence perception with different speaking styles | Participants achieved mean scores of 85.4%, 84.7%, 74.8%, 53.7%, 81.5%, and 80.3% correct for slow, normal, fast, whispered, emotional, and shouted speech, respectively. |
| Ren et al, 2018 (N=33) | AAI: M=4.53, SD=6.60 years  DCI: M=7.5, SD=3.0 years | Open-set word recognition in speech-spectrum-shaped noise and in four-talker babble (The LNT) | Participants’ word recognition scores reduced with increasing SNR, more difficult lists and four-talker babble. |
| Tao et al, 2018 (N=16) | Age: M=9.1, R=7-14 years  DCI: M=5.1, R=2-13 years | Sentence perception in steady-state noise and in competing speech | Participants performed better in steady-state noise than in competing speech. |
| Hong et al, 2019 (N=60) | Three groups of participants in kindergarten, primary and high school | Consonant recognition in quiet  Sentence perception in quiet | Children in kindergarten recognized consonant and sentence the best, followed by children in primary school and high school. |

Abbreviations: AAI: Age at Implant; DCI: Duration of CI use; M: Mean; N: The number of participants; R: Range; SD: Standard deviation; SNR: Signal-to-Noise Ratio; The LNT: the Standard-Chinese version of the Lexical Neighborhood Test; The MESP test: the Mandarin Early Speech Perception test; The MPSI test: The Mandarin Pediatric Speech Intelligibility test;

1 Only outcome measures and results related to speech perception were reported.

References:

Chen Y., Wong L.L., Chen F. & Xi X. 2014. Tone and sentence perception in young Mandarin-speaking children with cochlear implants. International journal of pediatric otorhinolaryngology, 78, 1923-1930.

Hong T., Wang J., Zhang L., Zhang Y., Shu H., et al. 2019. Age-sensitive associations of segmental and suprasegmental perception with sentence-level language skills in Mandarin-speaking children with cochlear implants. Research in developmental disabilities, 93, 103453.

Liu H., Liu S., Wang S., Liu C., Kong Y., et al. 2013. Effects of lexical characteristics and demographic factors on mandarin chinese open-set word recognition in children with cochlear implants. Ear and hearing, 34, 221-228.

Liu Q., Zhou N., Berger R., Huang D. & Xu L. 2013. Mandarin consonant contrast recognition among children with cochlear implants or hearing aids and normal-hearing children. Otology & Neurotology.

Ren C., Yang J., Zha D., Lin Y., Liu H., et al. 2018. Spoken word recognition in noise in Mandarin-speaking pediatric cochlear implant users. International journal of pediatric otorhinolaryngology, 113, 124-130.

Su Q., Galvin J.J., Zhang G., Li Y. & Fu Q.J. 2016. Effects of Within-Talker Variability on Speech Intelligibility in Mandarin-Speaking Adult and Pediatric Cochlear Implant Patients. Trends in hearing, 20.

Tao D., Liu Y., Fei Y., III J.G. & ... 2018. Effects of age and duration of deafness on Mandarin speech understanding in competing speech by normal-hearing and cochlear implant children. The J Acoust Soc Am, 144(2):EL131.

Zheng Y., Soli S.D., Meng Z., Tao Y., Wang K., et al. 2010. Assessment of Mandarin-speaking pediatric cochlear implant recipients with the Mandarin Early Speech Perception (MESP) test. International journal of pediatric otorhinolaryngology, 74, 920-925.

Zhu M., Fu Q.J., Galvin J.J., 3rd, Jiang Y., Xu J., et al. 2011. Mandarin Chinese speech recognition by pediatric cochlear implant users. International journal of pediatric otorhinolaryngology, 75, 793-800.
