# Supplementary material for: A Review of Speech Perception of Mandarin-Speaking Children With Cochlear Implantation
Source: Front Neurosci. 2021 Dec 14;15:773694. doi: 10.3389/fnins.2021.773694 (PMC8712552; doi:10.3389/fnins.2021.773694)
Supplement: Supplementary file 2 [file Table_2.docx]

Supplementary Table 2. Less-frequently examined factors that affect speech perception of children with unilateral CI

| Studies | Subject Characteristics | Outcome Measures^1^ | Analysis Method | AAD | AHL | CI | CL | DOD | Edu | Family | G | MDE | ST | UNHS |
| --- | --- | --- | --- | --- | --- | --- | --- | --- | --- | --- | --- | --- | --- | --- |
| Zheng et al, 2011  (N=39) | AAI: 1-2 years (n=4), 2-3 years (n=12), 3-4 years (n=12), 4-6 years (n=11) Tested at baseline, and 3, 6 and 12 months after CI | The MESP test | Pearson's correlation and χ2 test of independence | - | - | - | - | - | - | - | - | √* | - | - |
| Liu et al, 2013  (N=41) | AAI: M=2.0, SD=0.74, R=0.83-4.17 years | Mandarin consonant contrast perception | Linear regression | - | √ | - | - | - | - | - | - | - | - | - |
| Zhou et al, 2013^2^  (N=110) | AAI: M=3.96, SD=2.70, R=1.11-12.95 years | Lexical tone recognition in quiet | Step-wise linear regression | - | - | √ | - | - | √ | √ | - | - | √ | - |
| Chen et al, 2015^3^  (N=115) | Test Age: M=4.16, SD=1.05, R=2.50-7.09 years  AAI: M=2.67, SD=1.08, R=0.69-5.00 years | Overall speech perception^4^ | Structural equation modelling | - | - | - | - | - | - | - | - | - | - | √* |
| Liu et al, 2015  (N=33) | AAI: M=2.02, SD=0.89, R=0.5-3.83 years  Tested at baseline, 1, 3, 6, 9, 12, 18, and 24 months after CI | The LEAQ | ANOVA | - | - | - | √* | - | - | - | - | - | - | - |
| Tao et al, 2015^5^  (N=21) | ATT: M=10.8, R=6-16 years AAI: M=4.3, R=2-12 years (Prelingual group) | Lexical tone perception in quiet | Linear egression | - | - | - | - | √* | - | - | - | - | - | - |
| Lu & Qin, 2018^6^  (N=132) | AAI: M=3.4 SD=1.35 years Tested at baseline, 3, 6, 9, 12, 18, 24 months after CI | Early speech perception^7^ | Multiple linear and logistic regression | - | - | √ | - | - | - | √* | √ | - | - | - |
| Fan et al, 2020^8^  (N=52) | AAI: Median=1.25, R=0.83-5.66 years  Tested at baseline, 3, 6, 9, 12, 15, 18, 21 and 24 months after CI | Closed monosyllables and disyllables recognition  CAPQ | Generalized estimating equation | - | - | √ | - | - | - | - | √ | - | √* | - |
| Jiang et al, 2020  (N=100) | AAI: Median=4.0, R=3.0-7.0 years  Tested at 1months, 1, 2, and 3 years after CI | CAPQ | Mann–Whitney test | - | - | - | √ | - | - | - | √ | - | √* | - |

Abbreviations: AAD: Age at Diagnosis; AHL: Aided Hearing Level; ANOVA: Analysis of Variance; CAPQ: The Categories of Auditory Performance questionnaire; CI: Cochlear Implantation; CL: Community Location (rural or urban); DOD: Duration of Deafness; Edu: Educational variables; Family: family variables; G: Gender; LEAQ: The LittleEARS® Auditory Questionnaire; M: Mean; MDE: Mandarin Dialect Exposure; R: Range; ST: Speech Therapy; SD: Standard Deviation; The MESP test: the Mandarin Early Speech Perception test; UNHS: Having undergone universal newborn hearing screening;

‘**√***’ showed that this study examined the corresponding factor and found a significant correlation. ‘**√**’ showed that this study examined the corresponding factor but no significant correlation was found. ‘**-**’ showed that this study did not examine the corresponding factor.

Notes:

1. Only outcome measures and results related to speech perception were reported.
2. In Zhou et al. (2013), family variables included family size and household income; CI variables included processor type and speech processing strategy; educational variables included communication mode and duration of speech therapy.
3. In Chen et al (2015), 5 children used a HA in the non-implanted ear and were tested with both CI and HA on.
4. Overall speech perception referred to a single composite score was generated by combining results from MAIS, the MESP test and the MPSI test using the principal component analysis.
5. In Tao et al. (2015), post-lingually deafened children were tested as well, but no significance was found.
6. In Lu & Qin (2018), family variable was socioeconomic status; CI variables included side of implantation and Type of implant.
7. Early speech perception here referred to the IT-MAIS and the MESP test
8. In Fan et al (2020), 9 (18.4%) children used bilateral CIs and 11 (22.4%) children used bimodal stimulation. The CI variable was the side of CI

**Reference:**

Chen Y., Wong L.L., Zhu S. & Xi X. 2015. A Structural Equation Modeling Approach to Examining Factors Influencing Outcomes with Cochlear Implant in Mandarin-Speaking Children. PloS one, 10, e0136576.

Fan X., Sui R., Qi X., Yang X., Wang N., et al. 2020. Analysis of the developmental trajectory and influencing factors of auditory and speech functions after cochlear implantation in Mandarin Chinese speaking children. Acta oto-laryngologica, 140, 501-508.

Jiang F., Alimu D., Qin W. & Kupper H. 2020. Long-term functional outcomes of hearing and speech rehabilitation efficacy among paediatric cochlear implant recipients in Shandong, China. Disability and rehabilitation, 43, 2860–2865.

Liu H., Liu S., Kirk K.I., Zhang J., Ge W., et al. 2015. Longitudinal performance of spoken word perception in Mandarin pediatric cochlear implant users. International journal of pediatric otorhinolaryngology, 79, 1677-1682.

Liu Q., Zhou N., Berger R., Huang D. & Xu L. 2013. Mandarin consonant contrast recognition among children with cochlear implants or hearing aids and normal-hearing children. Otology & Neurotology, 34, 471–476.

Lu X. & Qin Z. 2018. Auditory and language development in Mandarin-speaking children after cochlear implantation. International journal of pediatric otorhinolaryngology, 107, 183-189.

Tao D., Deng R., Jiang Y., Galvin J.J., 3rd, Fu Q.J., et al. 2015. Melodic pitch perception and lexical tone perception in Mandarin-speaking cochlear implant users. Ear and hearing, 36, 102-110.

Zheng Y., Soli S.D., Tao Y., Xu K., Meng Z., et al. 2011. Early prelingual auditory development and speech perception at 1-year follow-up in Mandarin-speaking children after cochlear implantation. International journal of pediatric otorhinolaryngology, 75, 1418-1426.

Zhou N., Huang J., Chen X. & Xu L. 2013. Relationship between tone perception and production in prelingually deafened children with cochlear implants. Otology & neurotology, 34, 499-506.
